# The pain trajectory of juvenile idiopathic arthritis (JIA): translating from adolescent patient report to behavioural sensitivity in a juvenile animal model

**DOI:** 10.1186/s12969-019-0360-3

**Published:** 2019-08-27

**Authors:** Annastazia E. Learoyd, Debajit Sen, Maria Fitzgerald

**Affiliations:** 1 Department of Neuroscience, Physiology & Pharmacology, University College London, London, UK; 2 Arthritis Research UK Centre for Adolescent Rheumatology, University College London, London, UK

**Keywords:** Pain, JIA, Inflammation, Joint, Translation, Animal model, Arthritic activity, Child, Adolescent

## Abstract

**Background:**

While pain is a common symptom in JIA patients, it remains unclear why some JIA patients develop ongoing or persistent pain. Complex clinical and social settings confound analysis of individual factors that may contribute to this pain. To address this, we first undertook a retrospective analysis of pain reports in a JIA patient cohort with the aim of identifying potential factors contributing to persistent pain. We then carried out an experimental laboratory study, using joint inflammatory pain behaviour in rodents, to validate the role of these factors in the onset of persistent pain under controlled conditions.

**Methods:**

***Patients:*** Retrospective analysis of anonymised pain visual analogue scale (VAS) scores and accompanying clinical scores from 97 JIA patients aged 13–19 (mean: 16.40 ± 1.21) collected over 50 weeks. ***Rats:*** Experimental study of pain behaviour following intra-articular microinjection of complete Freund’s adjuvant (CFA) in adolescents (*n* = 25) and young adults (*n* = 43). Some animals (*n* = 21) had been previously exposed to joint inflammation in infancy or adolescence.

**Results:**

***Patients:*** Cluster analysis of patient pain VAS scores revealed three trajectories over 50 weeks: consistently low pain (*n* = 45), variable pain (*n* = 30) and persistently high pain (*n* = 22). Number of actively inflamed joints did not differ in the three groups. High pain at a single visit correlated with greater physician global assessment of disease activity, while a high pain trajectory over 50 weeks was associated with more limited joints but fewer actively inflamed joints. ***Rats:*** Rodents administered ankle joint CFA also exhibit low, medium and high joint pain sensitivities, independent of joint inflammation. Prolonged inflammatory pain behaviour was associated with high background pain sensitivity, following joint inflammation at an earlier stage in life.

**Conclusions:**

Both JIA patients and rodents differ in their individual pain sensitivity independent of the concurrent joint inflammation. Using experimental animal models allows us to isolate physiological factors underlying these differences, independently of social or clinical factors. The results suggest that a history of prior arthritic activity/joint inflammation may contribute to high pain sensitivity in adolescents with JIA.

**Electronic supplementary material:**

The online version of this article (10.1186/s12969-019-0360-3) contains supplementary material, which is available to authorized users.

## Background

Juvenile idiopathic arthritis (JIA) is a heterogenous group of inflammatory arthritides affecting 1 in 1000 children in the UK [1]. At least 70% of children with JIA report regular incidences of pain [2–4] and patients can continue to experience pain up to 30 years after JIA onset [5]. Chronic pain correlates with disability [2] and quality of life [6] in these patients; yet the prevalence of pain has barely improved despite increasing awareness and improving therapeutics [6, 7].

Recent studies show that only a subset of children with JIA (10–20%) develop persistent pain over 5 years from disease onset [8–10]. These findings suggest that pain sensitivity may vary in JIA patients and that certain biological and psychosocial factors may contribute to this sensitivity and the development of persistent pain. Age, [8–11] sex, [8, 12, 13] JIA subtype and arthritic activity, [10–12], disease duration, [9] symptoms of depression or anxiety, [3, 13, 14] stressful events [4], and dysfunctional health beliefs [15, 16] have all been implicated as possible contributing factors. However, evidence for the role of these factors in the development of pain is mixed. For example, the role of arthritic activity is disputed with some studies finding that the presence of active arthritis had no effect on pain sensitivity [13, 17] and others finding that increased numbers of inflamed joints are linked to a more persistent pain state in the following days [3] to years [8, 10].

Understanding the factors determining pain trajectory is crucial to furthering our understanding of JIA pain, but assessing the relative contribution of individual factors is extremely difficult in patient cohorts. Animals models provide an alternative approach – allowing contributing factors to be assessed in a controlled setting [18]. Age appropriate models of joint inflammation or arthritis allow individual factors to be adjusted and their influence on inflammatory pain in juveniles quantified, while also controlling for confounding variables such as disease activity, environment, genetic risk factors and maternal influences.

In the first part of this study we have undertaken a retrospective analysis of pain reports in a JIA patient cohort with the aim of identifying biological factors that explain reports of persistent pain. In the second part we have used an experimental rodent model of joint inflammatory pain, to validate the role of these factors in determining pain sensitivity in these models.

## Methods

### Part 1: patient cohort

#### Study design

This part of the study is a retrospective analysis of data collected from patients with JIA and their physicians at clinic visits to University College London Hospital. The data follows an observational study design examining factors influencing pain in a cross-section of adolescents with JIA.

#### Study population and data collection

Study participants attended routine clinical appointments at University College London Hospital between 2014 and 2018. At each appointment, patients completed a questionnaire which included the 10 cm pain Visual Analogue Scale (VAS), the Childhood Health Assessment Questionnaire (CHAQ) and a 10 cm patient general evaluation VAS (PGE). Concurrently, physicians completed a 10 cm physician global assessment VAS (PGA) and recorded the number of active (inflamed), swollen and limited joints. Patient characteristics (age, gender, age of JIA onset, JIA subtype, medications) were noted for each appointment. All data was anonymised prior to patient selection and statistical analysis.

Disease activity markers erythrocyte sedimentation rate and c-reactive protein levels were only available for 43% of clinic visits because blood sampling is only routine practice in patients receiving disease-modifying antirheumatic drugs (DMARDs) or biologics and because the data was only available if blood sampling coincided with a clinic visit. As such these factors could not be included in the analysis without losing statistical power. Information on disease markers for each pain trajectory is available in Additional file 1: Table S1.

Physician reported outcomes are drawn from evaluations made during clinical appointments. Several rheumatologists contributed to the data with an agreed consensus between physicians. Intra-investigator variation was not assessed due to the retrospective nature of this study.

#### Patient selection

In total, 282 patients with JIA attended the clinic for at least 1 year. Sixteen were excluded due to a change to a non-JIA diagnosis. The remaining patients made 1714 clinic visits overall with varying intervals between appointments. Completion of the patient questionnaire varied (67.3% completed). Physician-reported measures (PGA and joint counts) were reported at 80.4% of visits. Patient characteristics are shown in Table 1.

**Table 1:** Patient characteristics at study onset

	All patients	Trajectory:			Statistical analysis	
		Low pain	Variable pain	High pain	F value/Chi-Square	*P* value
No. of patients	97	45 (46.4%)	30 (30.9%)	22 (22.7%)		
Age at JIA onset	9.62 (4.86)	9.51 (4.71)	9.96 (4.88)	9.40 (5.31)	0.11	0.90
Age at study onset	16.40 (1.21)	16.19 (1.18)	16.46 (1.16)	16.76 (1.31)	1.67	0.19
Years since JIA onset	6.78 (5.17)	6.68 (4.97)	6.49 (5.33)	7.36 (5.55)	0.19	0.83
Sex:					8.05	***0.018***
Female	55 (56.7%)	24 (53.3%)	13 (43.3%)	18 (81.8%)		
Male	42 (43.3%)	21 (46.7%)	17 (56.7%)	4 (18.2%)		
Weeks between:						
Visit 1 and 2	24.12 (4.11)	24.24 (4.16)	24.30 (4.50)	23.64 (3.55)	0.20	0.82
Visit 1 and 3	49.11 (5.74)	49.27 (4.92)	48.63 (6.91)	49.45 (5.77)	0.16	0.86
JIA subtype:					11.77	*0.067*
Polyarticular course	54 (55.7%)	21 (46.7%)	16 (53.3%)	17 (77.3%)		
Oligoarticular	6 (6.2%)	4 (8.9%)	1 (3.3%)	1 (4.5%)		
Enthesitis Related	32 (33.0%)	15 (33.3%)	13 (43.3%)	4 (18.2%)		
Systemic	5 (5.2%)	5 (11.1%)	0 (0.0%)	0 (0.0%)		
JIA activity markers:						
PGA (cm)	2.40 (2.52)	1.40 (2.11)	3.11 (2.73)	3.47 (2.31)	7.64	***0.001***
No. of active joints	0 (0–2)	0 (0–1)	1 (0–1)	0 (0–2)	2.06	0.13
Medications:						
No. taking DMARDs	67 (69.1%)	27 (60.0%)	22 (73.3%)	18 (81.8%)	3.66	0.16
No. taking Biologics	40 (41.2%)	18 (40.0%)	12 (40.0%)	10 (45.5%)	0.21	0.90
No. taking Steroids	16 (16.5%)	4 (8.9%)	7 (23.3%)	5 (22.7%)	3.53	0.17

Data is presented as mean (standard deviation) for continuous data, number (% of patients with trajectory type) for categorical data or median (interquartile range) for discrete data (e.g. joint count). Comparisons between trajectories which reach significance (*p* < 0.05) are indicated in bold. PGA = Physician global assessment VAS. DMARDs = Disease-modifying antirheumatic drugs

**Fig. 1:**
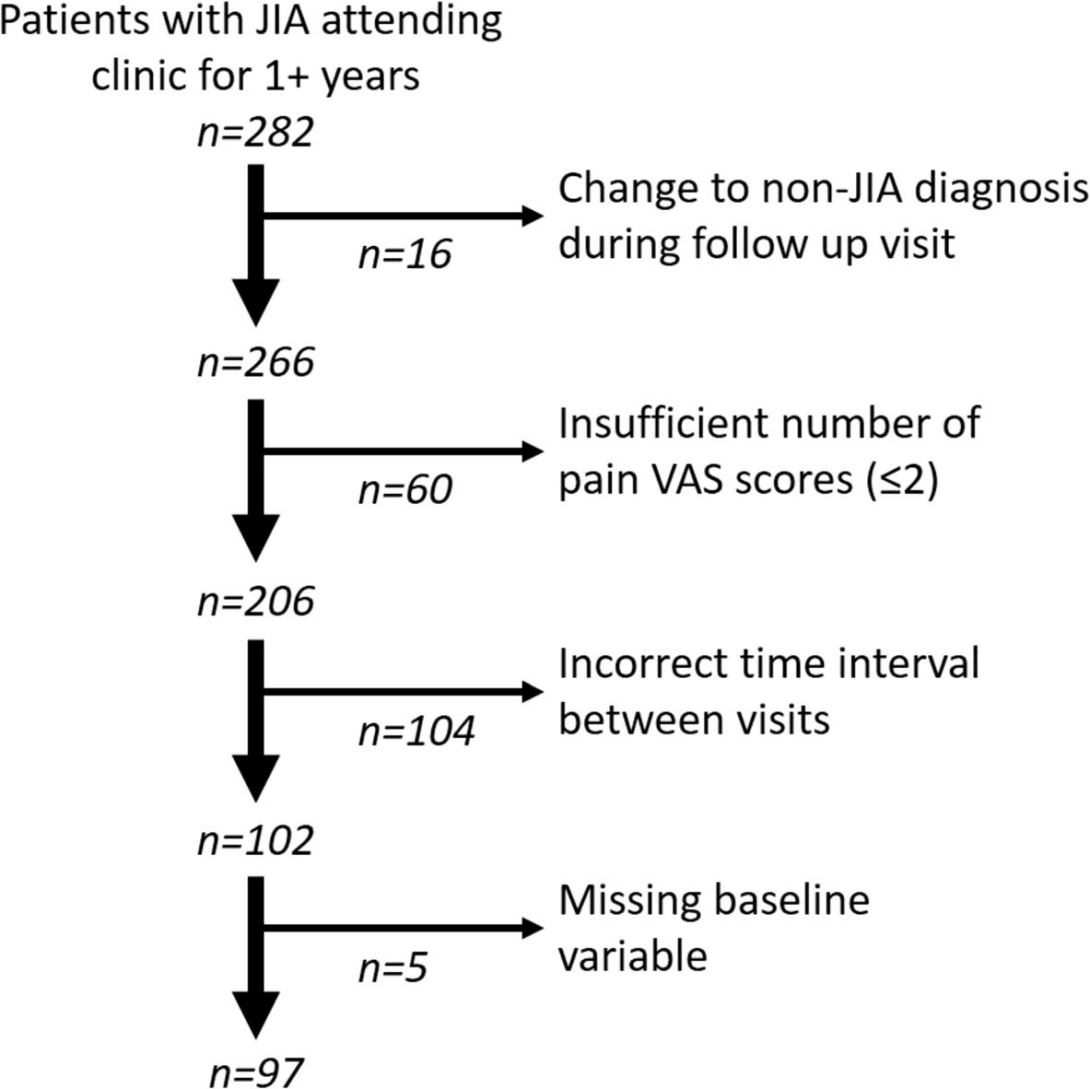
Exclusion criteria used for the selection of JIA patients for the analysis of pain trajectories over 50 weeks and the resultant number of patients included in this analysis

Only patients with 3 pain VAS scores obtained at 25 ± 7 week intervals were included in the analysis. Data collection was opportunistic, leading to the following exclusions: 60 patients with an insufficient number of pain VAS scores, 104 patients with incorrect time intervals between visits, and 5 patients due to missing baseline variables. The exclusion criteria are shown in Fig. 1 and the characteristics of excluded patients are shown in Additional file 1: Table S2.

In total, 97 patients were included in this analysis (Fig. 1), aged 13.77–19.44 years old (mean: 16.40 ± 1.21) with the age of JIA diagnosis ranging from 0.97–17.00 years old (mean: 9.62 ± 4.86). Disease duration at study onset ranged from 0.22–17.45 years (mean: 6.78 ± 5.17). 56.7% of patients were female. Patients most commonly had JIA with a polyarticular course (55.7%). This subset was made up of patients with either extended oligoarticular JIA (37.04%) or polyarticular JIA (62.96%).

#### Statistical analysis

Analyses were performed using SPSS, version 25. Patient pain trajectories were identified using hierarchical cluster analysis. Distinctions between trajectories were identified using repeated measures two-way ANOVA with Bonferroni post-hoc analysis. Characteristics of patients with each trajectory type were compared using either one-way ANOVA or Chi-Square test, depending on the type of data.

The influence of current arthritic activity on pain was determined using linear regression assessing the correlation of physician-reported measures with pain at a single clinic visit. PGA and the number of active joints for each trajectory type were also compared using one-way ANOVA with Bonferroni post-hoc analysis.

Factors associated with each trajectory type in patients were identified using multinomial logistic regression. Physician-reported measures collected at study onset and patient characteristics were included in this analysis. In addition, the association of active, swollen or limited joints (or a combination of the three) with trajectory type was examined using Fisher’s Exact test.

### Part 2: rodent model of monoarthritis

#### Study design

This part of the study examined factors, highlighted in the analysis of patient data, in an experimental animal model of monoarthritic pain. This allowed us to assess their contribution to inflammatory pain sensitivity in the absence of confounding factors.

Animal experiments were performed under a project license from the UK Home Office. Male and female Sprague-Dawley rats were bred and maintained in-house at UCL under standard conditions (21-23^o^c, 12h light-dark cycle) with unlimited food and water. Handling and maternal separation of pups were kept to a minimum and animals were exposed to the same standard caging, handling and diet throughout.

Randomisation using a random number generator was utilised wherever possible, including group allocation and animal order while undergoing procedures. Blinding of group assignment was not possible due to the clear differentiation in joint inflammation between experimental and control animals. Full details of animal numbers are provided in Additional file 1: Table S3 and Table S4.

Reporting on this study is based on the ARRIVE Guidelines for Reporting Animal Research [19].

#### Monoarthritis induction

Monoarthritis was induced by a microinjection of complete Freund’s adjuvant (CFA; Sigma-Aldrich, UK) into the intraarticular space of the left ankle joint. Animals were anaesthetised with isoflurane and a 30-gauge needle attached to a 100 μl Hamilton syringe was inserted into the ankle joint from the posterior lateral aspect. The minimum volume of CFA required to produce acute arthritic inflammation was administered, namely 2, 10, or 20 μl CFA for animals aged postnatal day (P)8, P21 and P40 respectively (human development equivalent: neonate, adolescent and young adult respectively). Control animals were administered sterile saline using the same procedure. In some animals, intraarticular injections of CFA were administered at two ages: either P8 or P21 and again at P40 (*n* = 12/sex). Control animals received CFA for the first time at P40 (*n* = 12/sex) or saline (*n* = 12/sex). Three animals receiving CFA twice were removed from the study due to excessive joint inflammation and pain behaviour. Two animals were excluded due to no pain behaviour post-CFA.

#### Behavioural testing

In all animals, mechanical withdrawal threshold, weight bearing, and joint diameter of the ipsilateral hindlimb were measured prior to CFA/saline injection (baseline) and regularly (every 1–4 days) up to 24 days post-injection. Calibrated von Frey hairs were applied to the paw and the mechanical withdrawal threshold was determined using the up-down method as described elsewhere [20]. Thresholds were transformed into a percentage change from baseline.

Hindlimb weight bearing was measured using an incapacitance meter (Churchill Electronic Services) which measures the weight supported by each hindlimb of a stationary animal. Three readings collected over 1 min were averaged and the weight borne by the ipsilateral limb was expressed as a percentage of the weight borne across both hindlimbs.

Ankle joint diameter was assessed as an indicator of the extent of joint inflammation. This was measured using callipers while the animal was standing. The joint was measured across the widest point–the malleoli produced by the fibula and tibia. Joint diameters were transformed into a percentage change from baseline.

#### Statistical analysis

Analyses were performed using SPSS, version 25. Sensitivities to inflammatory pain were identified using hierarchical cluster analysis. Distinctions between different sensitivities were identified using repeated measures two-way ANOVA with Bonferroni post-hoc analysis. The distribution of male and females across pain sensitivities was examined using a Chi-Square test. The influence of current joint inflammation on pain sensitivity was determined using a linear regression assessing the correlation of joint diameter with % reduction in mechanical threshold 3 days after the induction of monoarthritis as well as a comparison of joint diameter across 21 days in animals with different pain sensitivities using repeated measures two-way ANOVA with Bonferroni post-hoc analysis.

To assess the effect of prior monoarthritis on pain sensitivity, animal groups were compared using two-way ANOVAs with Bonferroni post-hoc analysis. Comparison of weight bearing deficits present prior to the second bout of monoarthritis (at baseline) were made using one-way ANOVA with Bonferroni post-hoc analysis.

## Results

### Part 1: pain in adolescent JIA patients

#### Pain in JIA patients follows one of three trajectories over 50 weeks

**Fig. 2:**
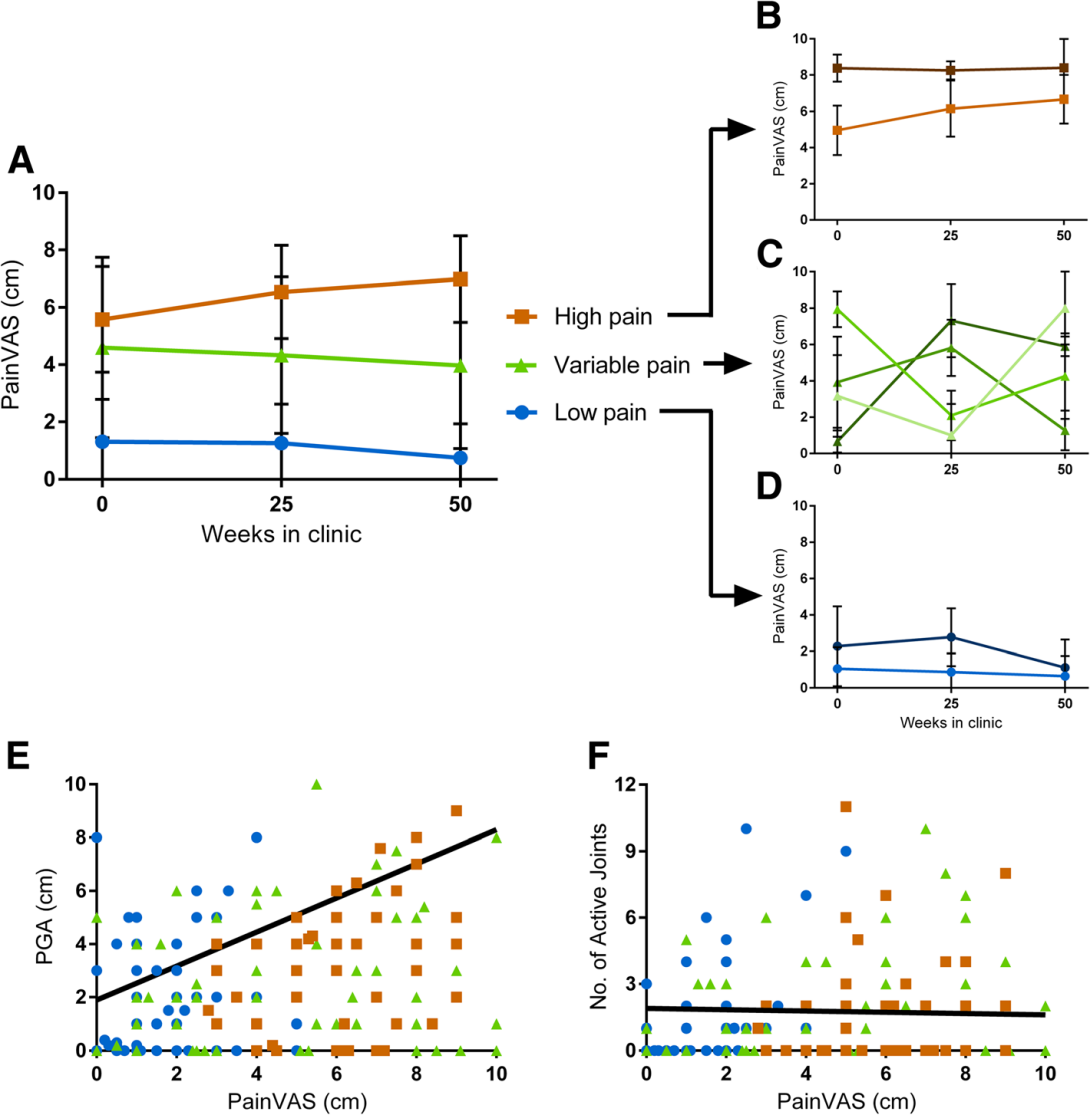
Cluster analysis of Pain VAS from JIA patients revealed several distinct clusters (**a**). These could be separated into 3 functional groups based on pain trajectory: patients with low levels of pain (*n* = 45, blue), patients with consistently high levels of pain (*n* = 22, orange) and patients with varying levels of pain over the 50 week period (*n* = 30, green). Each of these functional groups consisted of a number of smaller clusters shown in B, C, and D which show the clusters forming patients with high pain (**b**), variable pain (**c**) and low pain (**d**) respectively. Investigating the influence of arthritic activity on pain across patients, pain at an individual visit was associated with physician global assessment (PGA, β = 0.53, *p* < 0.001, **e**) but not the number of active joints (β = − 0.04, *p* = 0.67, **f**). Lines of best fit shown account for other factors included in the regression model

Pain visual analogue scores (VAS) reported by patients varied considerably at each clinic visit (range: 0.0–10.0 cm for each visit; mean: 3.3 ± 2.9 cm, 3.4 ± 2.9 cm and 3.2 ± 3.2 cm for each consecutive visit). Despite this variability, pain VAS over the 3 clinic visits could be separated into 8 discrete clusters which were aggregated into 3 larger functionally distinct pain trajectories based on proximity between clusters (Fig. 2a): patients with consistently low levels of pain (typically < 3.0 cm; 46.4% of patients, Fig. 2d), patients with persistently high pain (typically ≥5.0 cm; 22.7%, Fig. 2b), and patients with varying pain levels (30.9% of patients, Fig. 2c) across the 3 clinic visits. This final group maintained average pain VAS of 5.1 ± 3.1 cm, 4.2 ± 2.9 cm and 4.1 ± 2.7 cm for each visit but consisted of patients with both large increases and decreases in pain between visits (as shown by the separate clusters in Fig. 2c).

Pain VAS significantly differed between the three trajectory groups across clinic visits (F_2,94_ = 193.21, *p* < 0.001). Disability (CHAQ) and quality of life (PGE) followed similar trajectories (Additional file 1: Figure S1), differing significantly between groups (CHAQ: F_2,74_ = 50.31, *p* < 0.001; PGE: F_2,90_ = 71.17, *p* < 0.001).

Patients in the three pain trajectories did not differ in patient characteristics except for sex: females were more prevalent in the high pain trajectory (Chi-square = 8.05, *p* = 0.018) compared to either the low or variable trajectories (Table 1).

#### Arthritic activity does not account for the differing pain sensitivities in patients

In the patient cohort, pain at any individual clinic visit was significantly associated with physician global assessment (PGA) (odds ratio [OR](95% confidence interval [95%CI]) = 1.90(1.61–2.23), β = 0.51, *p* < 0.001; Fig. 2e) but not with the number of active joints (OR(95%CI) = 0.98(0.83–1.16), β = − 0.02, *p* = 0.81; Fig. 2f), or swollen (OR(95%CI) = 1.05(0.88–1.24), β = 0.04, *p* = 0.61) or limited joints at that visit (OR(95%CI) = 1.02(0.96–1.09), β = 0.04, *p* = 0.46). This model examining variables influencing concurrent pain, explained a significant amount of the variance seen in pain VAS scores (*F* = 24.90, *p* < 0.001) but not all (*R*^2^ = 0.28).

#### A high pain trajectory in patients is associated with limited joints at study onset

Since pain is not associated with measures of current arthritic activity, we investigated the association of factors at study onset with pain trajectory. Average scores for PGA was significantly higher (F_2,94_ = 7.64, *p* = 0.001) in patients with variable (*p* < 0.05) or high (*p* < 0.01) pain compared to those with low pain (Table 1), whereas the number of active joints did not significantly differ between trajectories (F_2,94_ = 2.06, *p* = 0.13), a pattern similar to that seen when correlating pain and concurrent arthritis activity.

However, regression analysis assessing the influence of disease activity markers alongside patients characteristics showed that patients with a high pain trajectory were less likely to have active joints at the onset of this study, (OR(95%CI) = 0.62(0.40–0.97), *p* = 0.035) but they were more likely to have swollen joints (OR(95%CI) = 1.73(1.02–2.95), *p* = 0.044) or limited joints restricted in movement (OR(95%CI) = 1.57(1.06–2.32), *p* = 0.026) compared to patients with a low pain trajectory (Table 2). PGA continues to be associated with increased pain (OR (95%CI) = 1.44(1.03–2.02), *p* = 0.033).

**Table 2:** Association between patient characteristics/JIA indicators and pain trajectories

	Average (mean (SD), no. (%), median (IQR))			Association with … relative to low pain			
	Low pain	Variable pain	High pain	Variable pain		High pain	
	*n = 45*	*n = 30*	*n = 22*	OR (95%CI)	*P* value	OR (95%CI)	*P* value
*Patient characteristics*							
Age at JIA onset	9.51 (4.71)	9.96 (4.88)	9.40 (5.31)	1.00 (0.88–1.15)	0.98	1.08 (0.93–1.25)	0.34
Age at study onset	16.19 (1.18)	16.46 (1.16)	16.76 (1.31)	1.38 (0.86–2.21)	0.19	1.56 (0.87–2.79)	0.13
Female sex (ref: male)	24 (53.3%)	13 (43.3%)	18 (81.8%)	0.50 (0.14–1.82)	0.29	2.92 (0.53–16.15)	0.22
JIA subtype (ref: polyarticular course)							
Enthesitis Related	15 (33.3%)	13 (43.3%)	4 (18.2%)	1.35 (0.32–5.60)	0.68	0.59 (0.09–3.72)	0.57
Oligoarticular	4 (8.9%)	1 (3.3%)	1 (4.5%)	0.69 (0.06–8.19)	0.77	0.52 (0.03–8.79)	0.65
Medications (ref: not in use for each type)							
DMARDs	27 (60.0%)	22 (73,3%)	18 (81.8%)	1.35 (0.32–5.60)	0.40	3.72 (0.78–17.69)	*0.098*
Biologics	18 (40.0%)	12 (40.0%)	10 (45.5%)	1.01 (0.32–3.18)	0.98	1.90 (0.47–7.74)	0.37
Steroids	4 (8.9%)	7 (23.3%)	5 (22.7%)	1.11 (0.16–7.59)	0.91	0.68 (0.08–5.79)	0.73
*JIA activity markers at study onset*							
PGA	1.40 (2.11)	3.11 (2.73)	3.47 (2.31)	1.22 (0.90–1.64)	0.20	1.44 (1.03–2.02)	***0.033***
No. of:							
Active joints	0 (0–1)	1 (0–1)	0 (0–2)	0.73 (0.50–1.06)	*0.096*	0.62 (0.40–0.97)	***0.035***
Swollen joints	0 (0–0)	1 (0–2.25)	1 (0–3)	1.59 (0.99–2.57)	*0.057*	1.73 (1.02–2.95)	***0.044***
Limited joints	0 (0–1)	2 (0–3)	2 (0–5)	1.53 (1.04–2.26)	***0.033***	1.57 (1.06–2.32)	***0.026***

Data is described as mean (standard deviation (SD)) for continuous data, number (% of patients with trajectory type) for categorical data or median (interquartile range (IQR)) for discrete data (joint count). Factors which are significantly associated (*p* < 0.05) with variable or high pain are indicated in bold. PGA = Physician global assessment VAS. DMARDs = Disease-modifying antirheumatic drugs. OR (95%CI) = Odds Ratio (95% Confidence Interval)

Patients with variable pain were also more likely to have limited joints (OR (95%CI) = 1.53(1.04–2.26), p = 0.033) than patients with low pain (Table 2) and have a trend towards having less active joints (OR (95%CI) = 0.73(0.50–1.06), *p* = 0.096) and more swollen joints (OR (95%CI) = 1.59(0.99–2.57), *p* = 0.057). This trajectory was not associated with PGA (OR (95%CI) = 1.22(0.90–1.64), *p* = 0.20).

Analysis of the distribution of these three joint measures (active, swollen, limited) within patients of each trajectory type revealed that the pattern of joint types differed significantly between the low and variable/high pain trajectories with fewer patients with only active joints but more patients with swollen and limited joint in patients with a variable/high pain trajectory (Fisher = 21.82, *p* = 0.029; Additional file 1: Figure S2).

This data suggests that patients with high levels of pain have less active arthritis, but more symptoms associated with arthritis activity at or before the onset of the study.

### Part 2: pain in a rodent model of joint inflammation

The data above shows that while that active arthritis is less likely in patients with high pain, they are more likely to have swollen and limited joints, indicating that prior arthritic activity may contribute to their high pain trajectory. To test whether there is a causal relationship between current pain sensitivity and prior joint inflammation, in the absence of other extraneous factors, we next turned to an experimental model of monoarthritis in rodents.

#### Inflammatory pain behaviour in a rodent model also follows three pain trajectories not associated with arthritic activity

**Fig. 3:**
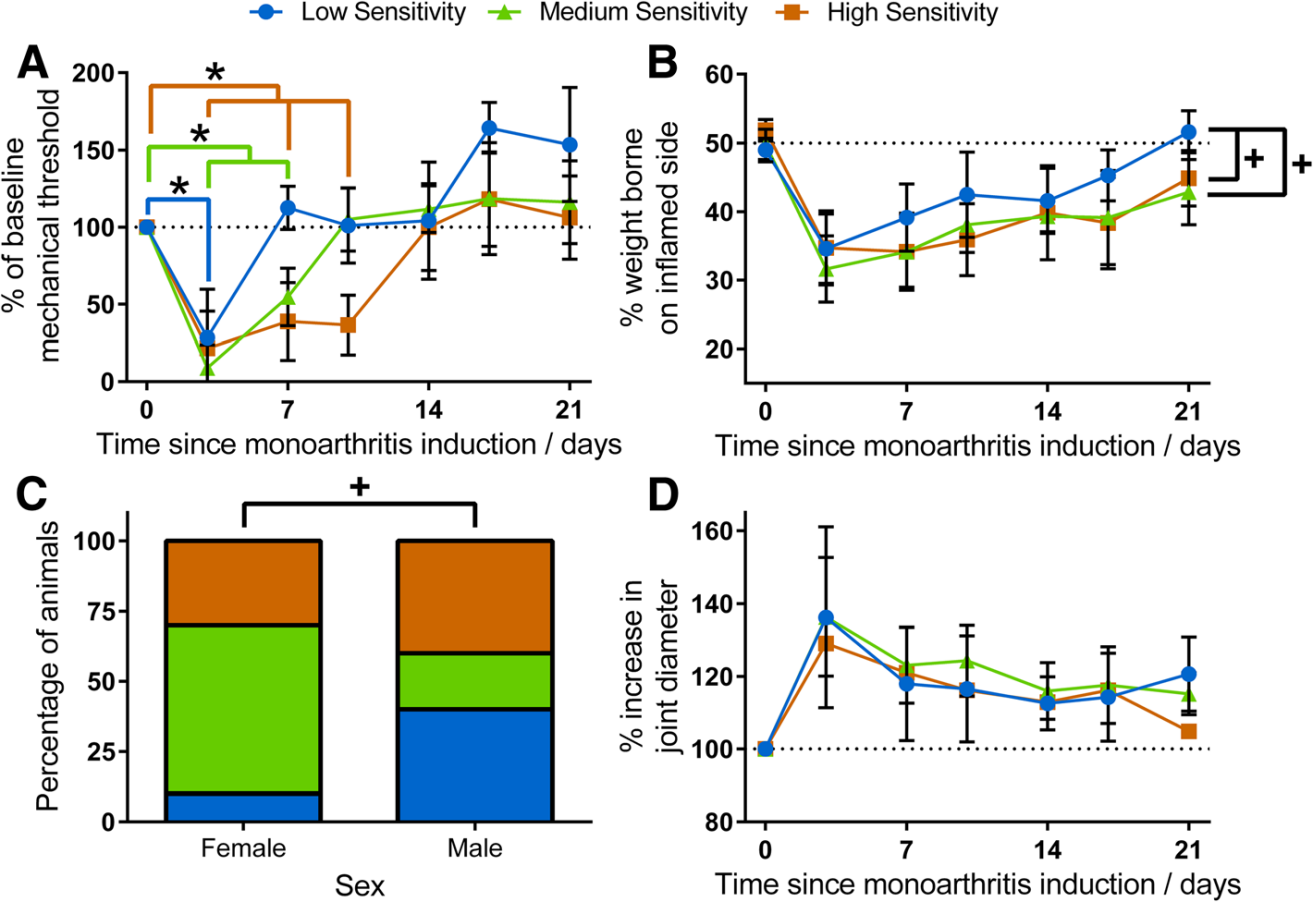
Cluster analysis of mechanical withdrawal threshold in rats aged P21 following monoarthritis revealed three distinct clusters (**a**). These could be related to differing pain trajectories with mechanical hypersensitivity (a reduction in mechanical withdrawal threshold) lasting different lengths of time (*p* < 0.001): 3 days for animals with low sensitivity to inflammatory pain (*n* = 7, blue; *p* < 0.001), 7 days for animals with a medium sensitivity to inflammatory pain (*n* = 9, green; *p* < 0.001), and 10 days for animals with a high sensitivity to inflammatory pain (*n* = 9, orange; *p* < 0.001). Weight bearing also differed between the sensitivity groups with animals with low sensitivity having a smaller reduction in weight bearing post-CFA compared to animals with medium or high sensitivity (*p* = 0.012, **b**). Females were more likely to have medium or high sensitivity (*p* < 0.001, **c**). These differing sensitivities to inflammatory pain were despite no difference in joint inflammation between the three groups (*p* = 0.89, **d**). * *p* < 0.05 between baseline mechanical threshold (0 day time-point) and indicated time-points for each sensitivity group as shown by the line colour. ^+^ *p* < 0.05 between indicated groups

Adolescent rats (P21) with complete Freund’s adjuvant (CFA) induced monoarthritis exhibited clear pain behaviour. This was measured by mechanical hypersensitivity for 7 days post-CFA injection (F_6,138_ = 53.01, *p* < 0.001) and reduced weight bearing on the inflamed limb for 21 days (F_6,138_ = 39.29, p < 0.001) with some variance. Based on mechanical withdrawal thresholds 3–10 days post-injection, animals could be separated into 3 clusters: those with a low (28.0%), medium (36.0%) or high (36.0%) sensitivity to pain. Animals in these three sensitivity groups also exhibited mechanical hypersensitivity after CFA injection for differing timescales (F_2,126_ = 6.65, *p* < 0.001, Fig. 3a): 3 days for animals with low pain sensitivity (0 vs. 3 days: *p* < 0.001), 7 days for animals with medium sensitivity (0 vs. 3–7 days: *p* < 0.001), and 10 days for animals with high pain sensitivity (0 vs. 3–10 days: *p* < 0.001).

Weight bearing on the inflamed limb also differed between sensitivity groups (F_2,21_ = 2.39, *p* = 0.012, Fig. 3b) with low pain sensitivity animals experiencing a smaller reduction in weight bearing post-injection compared to animals with a medium (vs. low: p = 0.012) or high pain sensitivity (vs. low: *p* = 0.044).

Females exhibited increased sensitivity with significantly more female rats having medium or high pain sensitivity compared to males (Chi-Square = 58.40, p < 0.001, Fig. 3c, Additional file 1: Table S3).

Arthritic activity did not account for pain sensitivity. Joint diameter (an indicator of the degree of joint inflammation) did not correlate with pain behaviour 3 days post-injection (F_1,9_ = 0.37, *p* = 0.56) and did not significantly differ between the three sensitivity types over 21 days post-injection (F_2,8_ = 0.12, *p* = 0.89, Fig. 3d). This data is consistent with the pattern seen in patients in Part 1.

#### Prior joint inflammation increases pain only in animals with high pain sensitivity

**Fig. 4:**
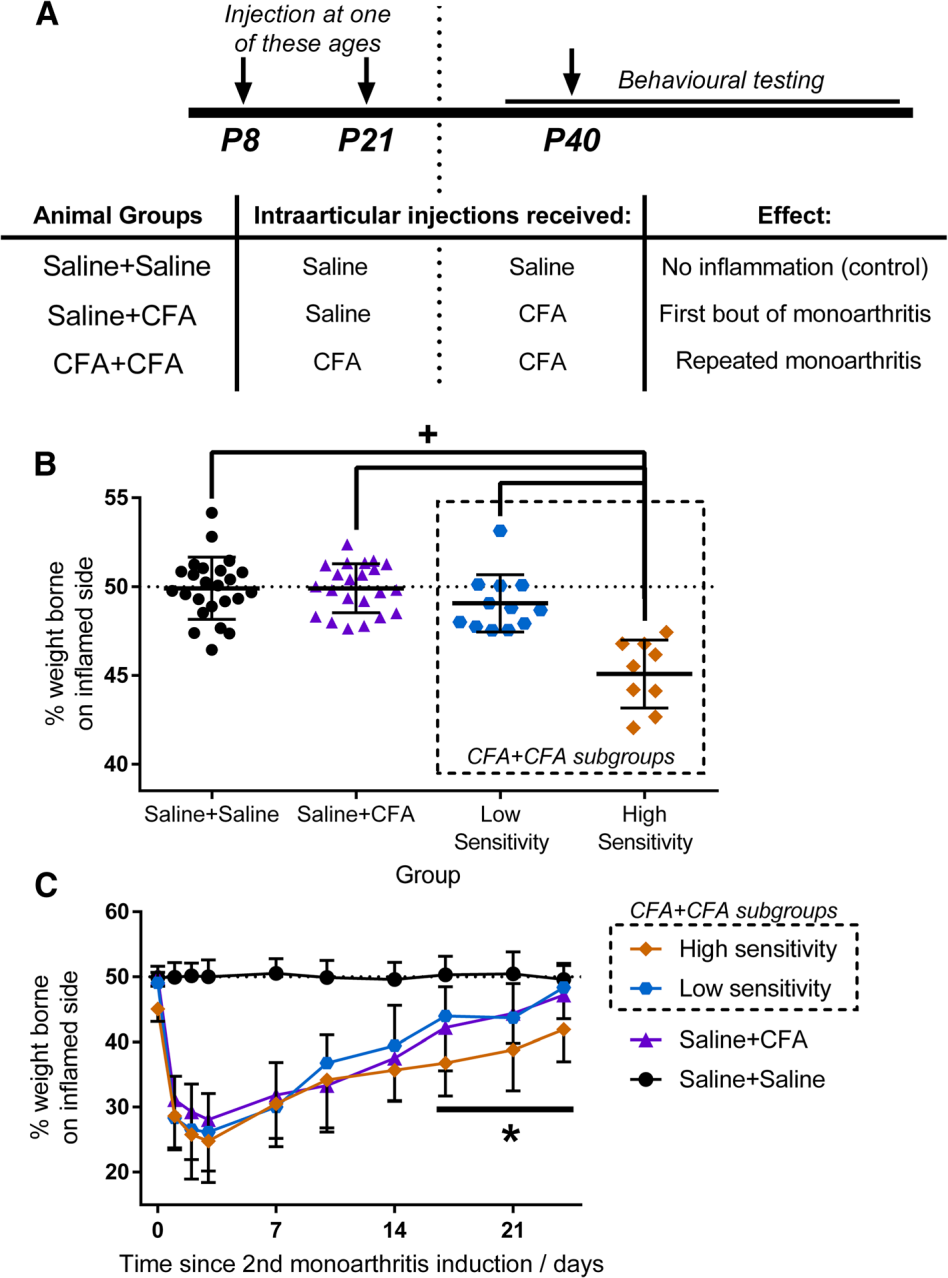
Pain behaviour was measured in animals receiving intraarticular injections of CFA or saline twice (at either P8/P21 followed by P40, **a**). CFA + CFA animals could be separated into animals with high or low pain sensitivity based on weight bearing measurements taken at baseline–prior to the second monoarthritis induction (subgroups indicated by dashed boxes). Animals with high pain sensitivity (*n* = 9, orange) have a significant reduction in baseline weight bearing compared to Saline+CFA (*n* = 22, purple) or Saline+Saline animals (*n* = 24, black) (*p* < 0.001, **b**) while those with a low pain sensitivity (*n* = 12, blue) did not. Following monoarthritis induction animals with high pain sensitivity experienced a prolonged reduction in weight bearing compared to animals with low pain sensitivity and Saline+CFA animals (*p* < 0.001, **c**). ^+^ *p* < 0.05 between indicated groups. * *p* < 0.05 between high sensitivity and low sensitivity/Saline+CFA at indicated time-points

To test whether pain trajectory is influenced by previous arthritic activity, pain behaviour was assessed in animals administered low dose joint CFA (or saline as a control) twice: once at P8 or P21, and again at P40 (Fig. 4a). Overall, pain behaviour in animals with two bouts of monoarthritis (CFA + CFA) was comparable to animals experiencing monoarthritis for the first time (Saline+CFA). Mechanical withdrawal threshold was reduced for 14 days post-injection in both groups (F_18,576_ = 8.43, *p* < 0.001; Saline+CFA vs. Saline+Saline controls: p < 0.001; CFA + CFA vs. Saline+Saline: *p* < 0.010). Weight bearing on the inflamed limb (F_18,576_ = 27.15, p < 0.001) was reduced for 21 days post-injection in Saline+CFA animals (vs. Saline+Saline: *p* < 0.001) and 24 days post-injection in CFA + CFA animals (vs. Saline+Saline: *p* < 0.05).

However, when CFA + CFA animals were separated into high or low pain sensitivity phenotypes based on their baseline levels of sensitivity prior to the second bout of CFA, significant differences emerged. The high sensitivity group were defined by their significantly lower baseline weight bearing compared to Saline+Saline and Saline+CFA animals (F_3.63_ = 22.02, *p* < 0.001; vs. Saline+Saline/Saline+CFA: p < 0.001; Fig. 4b) which animals with low sensitivity group did not have (vs. Saline+Saline/Saline+CFA: *p* > 0.050). Sex distribution did not differ the two groups (Chi Square = 0.40, *p* = 0.53, Additional file 1: Table S4).

The effect of a second CFA injection upon pain behaviour, differed significantly between the low and high sensitivity groups. Animals with high sensitivity experienced significantly longer lasting pain, measured as a reduction in weight bearing, after the second CFA injection (F_27,567_ = 19.09, p < 0.001, Fig. 4c) compared to low sensitivity or Saline+CFA animals, from 17 to 24 days post-injection (vs. low sensitivity/Saline+CFA: *p* < 0.05). Reductions in mechanical withdrawal threshold (F_21,441_ = 11.96, p < 0.001) and joint inflammation did not differ between groups (F_27,567_ = 5.76, p < 0.001).

This data shows that rodents, like human patients, vary in their sensitivity to joint inflammation. The data also shows that prior inflammatory activity does lead to enhanced monoarthritic pain, but only in those animals that have a higher sensitivity at baseline.

## Discussion

Here we have used a combination of JIA patient data and rodent inflammatory pain behaviour to identify and examine factors which contribute to persistent pain in children with JIA. The first part of the study identified 3 trajectories of pain over 50 weeks in adolescents with JIA. Pain trajectory was not dependent on current arthritic activity (as measured by active joint count), nor did other discrete disease-related variables (JIA subtype or medications administered) differ between trajectories. However, a different subjective measure of disease activity–the physicians’ assessment (PGA)–did correlate with pain reported at an individual visit. Evaluating factors related to each pain trajectory we found that, while active arthritis is less likely in patients with high pain, these patients had more swollen and limited joints suggesting that prior arthritic activity may influence the progression of pain.

This was explored in the second part of the study using an experimental animal model of joint inflammation to examine the role of prior inflammation in the development of persistent pain. Recurrent joint inflammation was established through the administration of CFA at two time-points. As predicted from the patient data, a prior bout of inflammation exacerbated pain, measured by weight bearing, but only in animals with higher baseline levels of pain sensitivity.

Divergent pain trajectories have been shown previously in JIA [8–10]. Studies describe a consistently low pain trajectory in many patients (≥50%) and a persistently high pain trajectory in 11.1% [8] or 17.9% [9] of patients, lower than reported here (24.2%). These previous studies focussed on pain in patients with new-onset JIA, whereas this is the first longitudinal study in adolescent patients with established JIA (average: 6.88 ± 5.21 years, range: 0.5–17.45 years), mapping pain levels across a year. Increased disease duration is the likely reason for the higher percentage of patients with high pain reported here and is consistent with studies measuring JIA pain at later time-points (up to 30 years), which describe persistent pain in 19% of patients [5, 21]. With the exception of the low and high pain trajectories described above, [8, 9] studies typically report pain which decreases over the first year from diagnosis before stabilising, [8–10, 14] a trajectory not prevalent here. Three patients in our variable pain trajectory had new-onset JIA (<1 year since diagnosis) and match this profile, but much of the group does not.

Psychosocial variables (ranging from depressive symptoms to health beliefs) were not included in this analysis due to the retrospective nature of the data collection. Despite this limitation, our data suggests that prior arthritic activity can exacerbate pain in individuals with high sensitivity to inflammatory pain. In patients, increased limited joint count was associated with variable and high pain trajectories, despite a lack of correlation between limited joints and pain reported at a single visit. Limited joint count is not typically included in analyses of factors influencing pain, indicating the novelty of this discovery. However, association does not necessarily indicate cause and it is hard to define the chronology of arthritic activity and pain. Indeed, pain within 6 months of JIA onset has been associated with increased disease severity, notably polyarticular disease and continued pain/disability at 5 or 8 years after JIA onset [10, 22].

Severe JIA has been differentiated into those requiring more medication but achieving control of arthritic activity and pain and those who, despite intensive treatments and comparable arthritic activity, continue to report pain and reduced quality of life [10]. This suggests that there are individual sensitivities to pain beyond arthritis itself, a feature observed in other chronic pain conditions [23]. Research in this area has focussed upon differences in individual neural pain pathways due to genetic and epigenetic processes, alterations in brain networks concerned with reward, motivation/learning and descending modulatory control and ‘priming’ of the pain system by previous pain and trauma [24].

With variations present in the pain trajectories in JIA patients, cross-species studies can facilitate characterisation of the relationship between disease activity and pain and dissect out underlying mechanisms. Our results from rodents suggest that–even within a colony of Sprague Dawley rats from a single institution–there are differences in innate sensitivity to inflammatory pain leading to low or high pain trajectories in arthritis. Here we show that the relationship between high pain and prior arthritic activity is dependent on this sensitivity. Individual pain sensitivities in rodents have been described elsewhere, focussing on genetic differences between rodent strains [25–27] or between sexes [28]. Individual differences in the response to acute noxious stimuli in rats of the same strain have been reported previously, [29, 30] but differences in the pain behaviour trajectories following joint inflammation has not.

We have previously proposed that preceding painful events may exacerbate pain in JIA, [31] and here we provide the first evidence supporting this proposal. Pain priming in early life has been established in rodent models of surgical pain, cutaneous and visceral inflammation (reviewed in [32]), but this is the first demonstration that low doses of CFA–inducing transient joint inflammation in juvenile animals exacerbates pain behaviour in adult rats. This model can be used to further dissect the mechanisms underlying persistent JIA pain and its relationship with previous inflammatory pain exposure, particularly the role of neuroimmune interactions in central pain circuits, which may establish a prolonged state of central sensitization not related to current inflammation [18, 33].

## Conclusions

This work shows that both patients with JIA and rodents with experimental joint inflammation have individual sensitivities to arthritic inflammatory pain which is not related to current joint inflammatory status. A significant number of both JIA patients and rodent models have pain that is persistently higher or more prolonged than other individuals of the same age, sex and arthritic activity. The results support the hypothesis that prior, rather than current, arthritic activity may be an explanatory factor for those individuals with a high sensitivity to inflammatory pain.

## Additional file

Additional file 1: **Table S1.** JIA disease activity markers for each pain trajectory. **Table S2.** Patient characteristics of included and excluded patients. **Figure S1.** Disability and quality of life as reported by patients with different levels of pain. **Figure S2.** Ven diagrams examining proportions of patients with each type of joint count. **Table S3.** Animal numbers and the distribution of pain trajectories in female and male adolescent rats. **Table S4.** Animal numbers and characteristics of animal groups receiving one or two bouts of monoarthritis. (DOCX 353 kb)

## Data Availability

The datasets obtained and analysed during the current study are available from the corresponding author on reasonable request.

## Abbreviations

CFA: Complete Freund’s adjuvant
CHAQ: Childhood Health Assessment Questionnaire
DMARDs: Disease-modifying antirheumatic drugs
JIA: Juvenile idiopathic arthritis
PGA: Physician global assessment
PGE: Patient general evaluation
VAS: Visual analogue scale
