# Supplementary material for: The pain trajectory of juvenile idiopathic arthritis (JIA): translating from adolescent patient report to behavioural sensitivity in a juvenile animal model
Source: Pediatr Rheumatol Online J. 2019 Aug 27;17:60. doi: 10.1186/s12969-019-0360-3 (PMC6712651; doi:10.1186/s12969-019-0360-3)
Supplement: Supplementary file 1 — Table S1. JIA disease activity markers for each pain trajectory. Table S2. Patient characteristics of included and excluded patients. Figure S1. Disability and quality of life as reported by patients with different levels of pain. Figure S2. Ven diagrams examining proportions of patients with each type of joint count. Table S3. Animal numbers and the distribution of pain trajectories in female and male adolescent rats. Table S4. Animal numbers and characteristics of animal groups receiving one or two bouts of monoarthritis. (DOCX 353 kb) [file 12969_2019_360_MOESM1_ESM.docx]

# The pain trajectory of juvenile idiopathic arthritis (JIA): translating from adolescent patient report to behavioural sensitivity in a juvenile animal model

# Supplementary material

Annastazia E Learoyd^1^, Debajit Sen^2^, Maria Fitzgerald^1^

^1^Department of Neuroscience, Physiology & Pharmacology, University College London, UK and ^2^Arthritis Research UK Centre for Adolescent Rheumatology, University College London, UK

# METHODS

## Part 1: Patient cohort

|  | **All patients** | **Trajectory:** | | | **Statistical analysis** | |
| --- | --- | --- | --- | --- | --- | --- |
|  |  | **Low pain** | **Variable pain** | **High pain** | **F value/Chi-Square** | **P value** |
| Total no. of patients | 97 | 45 (46.4%) | 30 (30.9%) | 22 (22.7%) |  |  |
| ***Included disease activity markers*** | | |  |  |  |  |
| PGA (cm) | 2.40 (2.52) | 1.40 (2.11) | 3.11 (2.73) | 3.47 (2.31) | 7.64 | ***0.001*** |
| No. of: |  |  |  |  |  |  |
| Active joints | 0 (0-2) | 0 (0-1) | 1 (0-1) | 0 (0-2) | 2.06 | 0.13 |
| Swollen joints | 0 (0-2) | 0 (0-0) | 1 (0-2.25) | 1 (0-3.25) | 4.33 | ***0.016*** |
| Limited joints | 1 (0-2) | 0 (0-1) | 2 (0-3) | 2 (0-5) | 3.16 | ***0.047*** |
| ***Blood disease markers:*** | |  |  |  |  |  |
| ESR | 11.98 (18.10) | 8.27 (11.97) | 16.13 (17.02) | 16.67 (29.74) | 1.54 | 0.22 |
| *No. of patients* | *61* | *33* | *16* | *12* |  |  |
| CRP | 3.75 (6.99) | 2.66 (3.96) | 5.77 (10.72) | 4.24 (7.93) | 0.90 | 0.41 |
| *No. of patients* | *50* | *28* | *13* | *9* |  |  |

Table 1 – JIA disease activity markers for each pain trajectory

Data is described as mean (standard deviation) for continuous data, number (% of patients with trajectory type) for categorical data or median (interquartile range) for discreet data (no. of active joints). Statistical comparison between trajectory groups were made using either one-way ANOVA or Chi-Square test. The number of patients with available information on blood disease markers is shown beneath each marker. PGA=Physician global assessment VAS.

|  | **Included patients** | **Excluded patients** | | **Statistical analysis** | |
| --- | --- | --- | --- | --- | --- |
|  |  | **≤2 pain VAS scores** | **Incorrect time intervals** | **F value/Chi-Square** | **P value** |
| No. of patients | 97 | 60 | 104 |  |  |
| PainVAS at study onset | 3.29 (2.89) | 2.78 (3.30) | 3.40 (3.08) | 2.78 | *0.064* |
| Age at JIA onset | 9.62 (4.86) | 9.13 (4.55) | 9.00 (4.93) | 0.48 | 0.62 |
| Age at study onset | 16.40 (1.21) | 17.39 (1.57)* | 16.46 (1.34) | 10.51 | ***<0.001*** |
| Years since JIA onset | 6.78 (5.17) | 8.78 (4.83) | 8.15 (5.39) | 1.60 | 0.20 |
| Sex: |  |  |  | 2.47 | 0.29 |
| Female | 55 (56.7%) | 30 (50.0%) | 65 (62.5%) |  |  |
| Male | 42 (43.3%) | 30 (50.0%) | 39 (37.5%) |  |  |
| JIA subtype: |  |  |  | 8.14 | 0.23 |
| Polyarticular course | 54 (55.7%) | 25 (41.7%) | 61 (58.7%) |  |  |
| Oligoarticular | 6 (6.2%) | 9 (15.0%) | 11 (10.6%) |  |  |
| Enthesitis Related | 32 (33.0%) | 20 (33.3%) | 25 (24.0%) |  |  |
| Systemic | 5 (5.2%) | 6 (10.0%) | 7 (6.7%) |  |  |
| JIA activity markers: |  |  |  |  |  |
| PGA | 2.40 (2.52) | 1.63 (2.55) | 2.25 (2.63) | 1.78 | 0.17 |
| No. of active joints | 0 (0-2) | 0 (0-1) | 0 (0-2) | 1.15 | 0.32 |
| Medications: |  |  |  |  |  |
| No. taking DMARDs | 67 (69.1%) | 35 (58.3%) | 56 (53.8%) | 5.03 | *0.081* |
| No. taking Biologics | 40 (41.2%) | 19 (31.7%) | 41 (39.4%) | 1.53 | 0.47 |
| No. taking Steroids | 16 (16.5%) | 6 (10.0%) | 13 (12.5%) | 1.47 | 0.48 |

Table 2 – Patient characteristics of included and excluded patients

Data is described as mean (standard deviation) for continuous data, number (% of patients with trajectory type) for categorical data or median (interquartile range) for discreet data (no. of active joints). Statistical comparison between trajectory groups were made using either one-way ANOVA or Chi-Square test. PGA=Physician global assessment VAS. * sig vs. included patients in post-hoc analysis.

# RESULTS

## Part 1: Pain in adolescent JIA patients


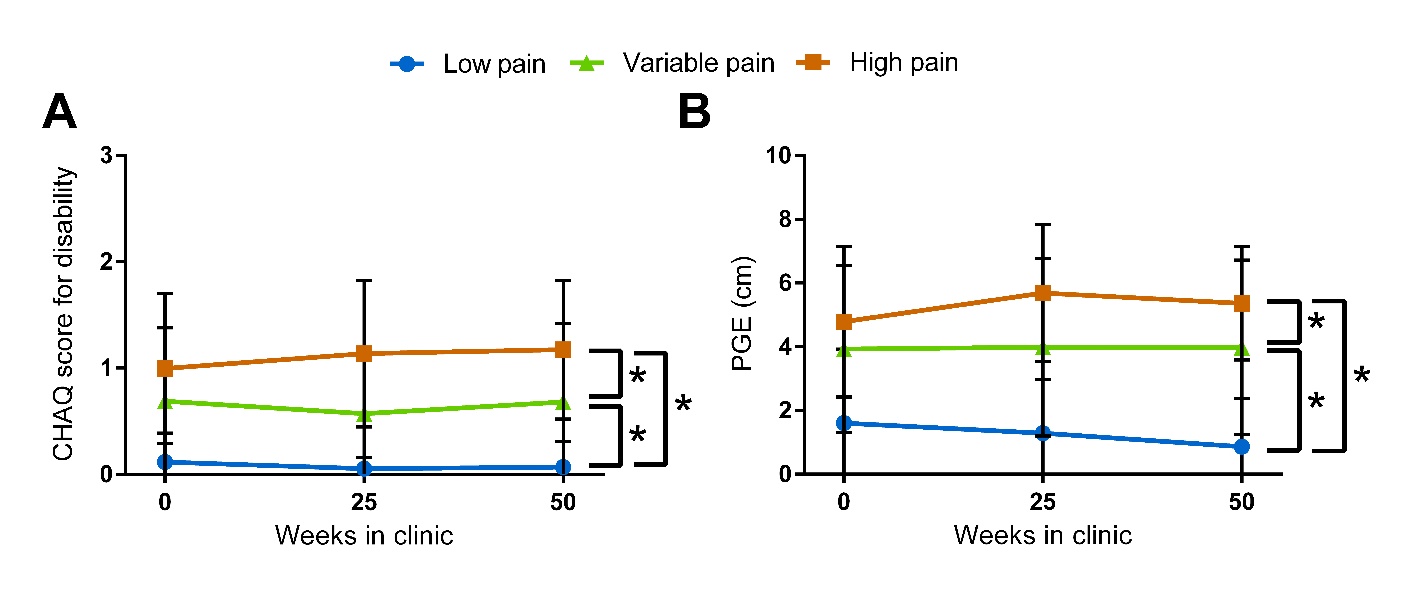


Figure 1 – Disability and quality of life as reported by patients with different levels of pain

Patients separated into low (n=45), variable (n=30) and high (n=22) pain trajectories via cluster analysis also experienced: **A**: differing levels of disability (as assessed by the Childhood Health Assessment Questionnaire [CHAQ]) (p<0.001) and **B**: varying levels of quality of life (as reported by the patient general evaluation VAS [PGE]) (p<0.001). * p<0.05 between indicated groups.


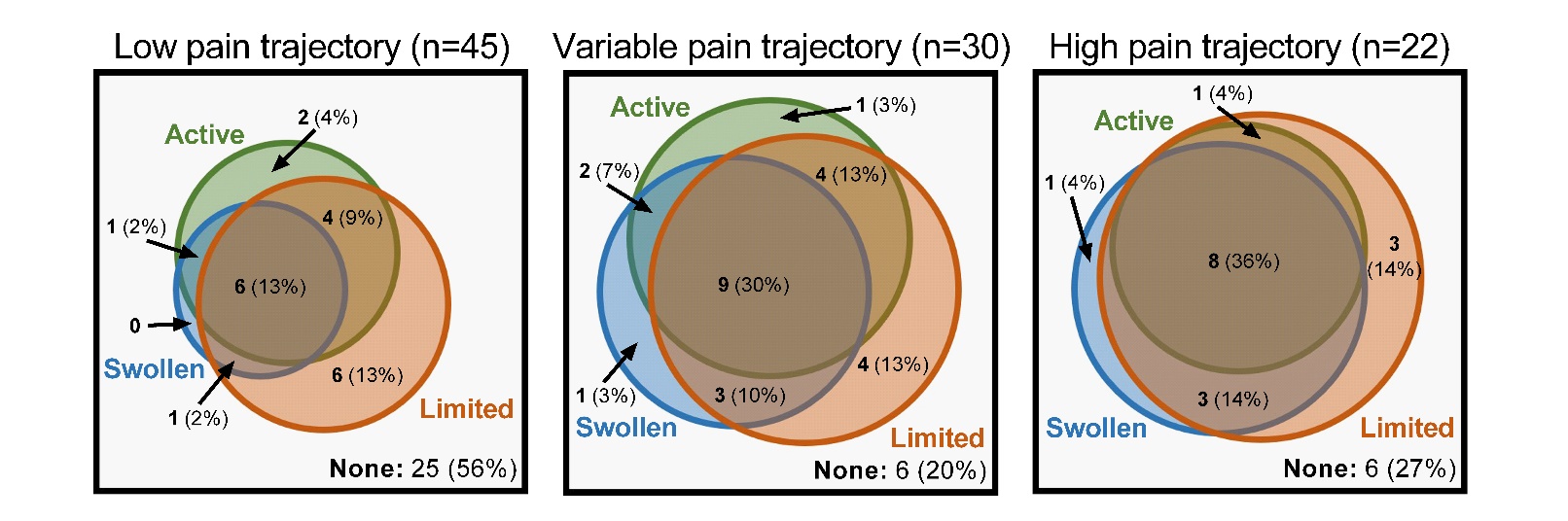


Figure 2 – Ven diagrams examining proportions of patients with each type of joint

Venn diagrams examining the proportion of patients with active (green), swollen (blue) or limited joints (orange), or a combination of the three, within each trajectory type revealed a reduced number of patients with only active joints and an increase in the number of patients with swollen and limited joints in the variable and high pain trajectory groups (p=0.029).

## Part 2: Pain in a rodent model of joint inflammation

|  | **All animals** | **Sex** | | **Statistical Analysis** | | |
| --- | --- | --- | --- | --- | --- | --- |
|  |  | **Female** | **Male** | **Chi-Square** | **P value** |  |
| No. of Animals | 25 | 10 | 15 |  |  |  |
| Trajectory: |  |  |  | 58.40 | ***<0.001*** |  |
| Low Sensitivity | 7 (28.0%) | 1 (10.0%) | 6 (40.0%) |  |  |  |
| Medium Sensitivity | 9 (36.0%) | 6 (60.0%) | 3 (20.0%) |  |  |  |
| High Sensitivity | 9 (36.0%) | 3 (30.0%) | 6 (40.0%) |  |  |  |

Table 3 – Animal numbers and the distribution of pain trajectories in female and male adolescent rats

Data is described as number (% of animals). Statistical comparison between trajectory groups were made using Chi-Square test.

|  | **All animals** | **Animal groups** | | | | |
| --- | --- | --- | --- | --- | --- | --- |
|  |  | **Saline+Saline** | **Saline+CFA** | **CFA+CFA** | CFA+CFA subgroups | |
|  |  |  |  |  | **Low Sensitivity** | **High Sensitivity** |
| No. of animals | 67 | 24 | 22 | 21 | 12 | 9 |
| Age of onset: |  |  |  |  |  |  |
| P8 | 35 (52.2%) | 12 (50.0%) | 11 (50.0%) | 12 (57.1%) | 8 (66.7%) | 4 (44.4%) |
| P21 | 32 (47.8%) | 12 (50.0%) | 11 (50.0%) | 9 (42.9%) | 4 (33.3%) | 5 (55.6%) |
| Sex: |  |  |  |  |  |  |
| Female | 34 (50.7%) | 12 (50.0%) | 11 (50.0%) | 11 (52.4%) | 7 (58.3%) | 4 (44.4%) |
| Male | 33 (49.3%) | 12 (50.0%) | 11 (50.0%) | 10 (47.6%) | 5 (41.7%) | 5 (55.6%) |
| Pain behaviour at baseline: | |  |  |  |  |  |
| % Weight bearing on inflamed limb | 49.11 (2.28) | 49.91 (1.74) | 49.91 (1.38) | 47.35 (2.64) | 49.06 (1.61) | 45.07 (1.92) |
| 50% log mechanical threshold | 1.12 (0.11) | 1.13 (0.10) | 1.10 (0.09) | 1.12 (0.13) | 1.13 (0.13) | 1.12 (0.15) |

Table 4 – Animal numbers and characteristics of animal groups receiving one or two bouts of monoarthritis

Data is described as mean (standard deviation) for continuous data or number (% of animals within group). Age of onset refers to the age monoarthritis was first induced: either postnatal day 8 or 21 (P8 or P21).
